# Modelling fundamental diagrams according to different water film depths from the perspective of the dynamic hydraulic pressure

**DOI:** 10.1038/s41598-020-63381-1

**Published:** 2020-04-16

**Authors:** Mingwei Liu, Matunaga Chiaki, Yoshinao Oeda, Tomonori Sumi

**Affiliations:** 1 Department of Engineering, Shanghai Ocean University, Shanghai, People’s Republic of China; 2 Department of Urban and Environment Engineering, Kyushu University, Kyushu, Japan

**Keywords:** Environmental social sciences, Natural hazards, Engineering, Mathematics and computing, Physics

## Abstract

In this paper, we propose enhanced fundamental diagrams based on different water film depths by considering the effects of hydroplaning using a physical method. Various factors are calculated to describe the total safe distance headway of main vehicle components. These factors include the driver reaction times, reaction distances, vehicle braking times, and vehicle braking distances corresponding to different water film depths. An excellent match is found between the computed braking distance, the braking time calculated using the proposed numerical model, and the results published in other papers. These calculations are performed to estimate the distance headway and quantitatively analyse the relationships between the speed, density, and water film depth. By using three road-specific parameters estimated by our proposed model, namely, the free-flow speed, jam density, and capacity flow, a link transmission model is developed to analyse the dynamic impact of the water film depth.

## Introduction

Urban flooding is one of the most severe types of flooding in terms of economic damage, health impact, and loss of life. It has become a significant challenge for the development and sustainability of coastal cities. Under more frequent extreme weather conditions, floods and inundations become more severe. For example, devastating torrential rains and subsequent flooding in western Japan left over 200 people dead in 2018. Evacuation can be an efficient solution for securing human safety in major disasters. One effective method for handling the consequences of hydrological disasters is to establish a prudent and comprehensive emergency management plan. Recently, several studies^1,2^, have attempted to model potential network capacity losses to estimate evacuation times from a probabilistic perspective. The objective of these studies was to estimate reliable evacuation times under realistic transportation network conditions.

Despite the fact that evacuation environments in flood conditions are typically characterised by great risk (road capacities and vehicle speeds may be reduced by water film depth), very few studies in the evacuation literature have explicitly considered this type of risk. A traffic model for representing traffic flow characteristics according to different water film depths is necessary to represent the traffic situation during emergency evacuations accurately. However, research in this field is very limited, especially regarding the evaluation of the influence of the water film depth. Therefore, it is essential to re-evaluate and enhance urban transportation planning models to capture the effects of flooding disasters related to water depth.

All traffic situations in the real world can be considered combinations of homogenous states, meaning they can be described by essential traffic flow characteristics (e.g., flow *Q*, speed *V*, and density *K*). Fundamental diagrams capture the relationships between these characteristics and play an essential role in traffic flow theory and transportation engineering. Various models have been proposed to represent the speed-density relationship mathematically. This relationship serves as the basis for fundamental diagramming. Original studies on fundamental diagramming involved linear relationships^3^, followed by significant advances in terms of improving simplified relationships^4–11^. These studies largely focused on developing empirically accurate single-regime models with small numbers of practically meaningful parameters. Sound mathematical models that accurately represent relationships can serve as a solid foundation for traffic flow analysis and efficient traffic control^12^.

To obtain relevant parameters and understand driver behaviour, the free-flow speed during free driving, variations in distance headway, and the mean speed of a convoy during bunched driving must be analysed. In the academic practice of determining the safe distance headway, data are required for the driver reaction time, safe distance under stationary conditions, and braking deceleration. In many reference books, such data refer to dry conditions and cover dynamic characteristics that differ from those under wet conditions. The international literature has indicated that weather and other factors affect not only road safety but also transportation in general^13^. In fact, several weather parameters may have a significant influence. The most common weather parameter considered in the literature is precipitation. Some researchers have concluded that rainfall of any intensity will adversely affect traffic operations. For example, one study^14^ indicated that the maximum highway volume and free flow speed are affected more strongly as the rainfall intensity increases. Compared with the case of normal weather, the maximum flow rate decreases by 15.7%, 19.1%, and 32.5% and the free flow speed decreases by 4.4%, 7.3%, and 10.6% in light rain, moderate rain and heavy rain weather conditions, respectively. Another study^15^ conducted research on the impact of rainfall on freeway traffic flow. The authors concluded that light rain reduces freeway capacity by 4% to 10% and that heavy rain reduces freeway capacity by 25% to 30%. Additionally, they noted that the presence of rain, regardless of its intensity, results in an approximate average decrease of 5% to 6.5% in driving speeds. Other weather parameters include air temperature, visibility, and wind speed.

The studies mentioned above concentrated on simple statistical methods based on observations. When data can be obtained easily, statistical methods yield accurate results and highlight the relationships between traffic stream characteristics and weather factors, such as precipitation. However, for standing water situations, it is very difficult to obtain observation data, meaning accurate models cannot be obtained through statistical methods, which is why studies on this topic are rare. Therefore, as an alternative to statistical measurement, we propose a physical approach to analyse the effects of the water film depth on road traffic. When a vehicle drives on wet roads, rainwater flows through its tire tread grooves, leading to an increase in hydrodynamic pressure. The resulting hydrodynamic force reduces the tire traction efficiency because it reduces the tire contact force. This leads to poor driving controllability and braking performance compared to the driving performance on dry roads. Simultaneously, this type of force in the horizontal direction opposes the advance of a vehicle and increases deceleration. Overall, the hydroplaning force has a significant effect on traffic flow. From this perspective, by using a physical approach, we can consider the mechanism of the water film influence between the tire treads and the road surfaces in a fundamental diagram.

The main contribution of this paper is the presentation of a physical method for modelling fundamental diagrams according to different water film depths from the perspective of hydrodynamic pressure. Using the proposed model, the essential parameters that constitute elementary knowledge for road surfaces under any water film depth can be obtained. The remainder of this paper is organised as follows. Section “Methods” discusses the modelling of the free flow speed and provides a description of the total safe distance headway of the main vehicle components, which consists of the driver reaction time, reaction distance, vehicle braking time, and vehicle braking distance considering the water film depth. These factors are analysed to estimate the distance headway and derive a quantitative relationship between the speed, density, and water film depth. This section also presents a  case study with data collection results for the free-flow speed, distance headway, and water film depth. In Section “Evacuation simulation via a LTM considering different water film depths”, a numerical simulation based on a link transmission model (LTM) is used to calculate the clearance times for evacuation based on the proposed model. The conclusion section contains our concluding remarks.

## Methods

### Modelling the free-flow speed

Detailed modelling procedures are provided in a separate publication^16^. In that study, a tire-sliding model was utilised to obtain the traction and friction forces between a road surface and a tire. An equation considering vehicle specifications and the water film depth was proposed to estimate the hydroplaning speed. By comparing observation data from Japan in 2009 to the estimated hydroplaning speeds, a safety factor was derived to generate a free-flow speed curve according to different water film depths.

### Estimation of the distance headway

#### Movement characteristics of vehicles during braking

The process of stopping involves significant deceleration to reduce the vehicle speed. The braking process is initiated when a driver activates the braking system of a vehicle by pressing the brake pedal. The braking process can be divided into two stages.

1. The driver reaction stage: During this period, the driver identifies a situation ahead that requires the vehicle to slow or stop and prepares to take immediate action.
2. The braking stage: During this period, the brake pedal is pressed to avoid a collision.

The displacement of the stopping process can be calculated using Eq. 1.

1 $${S}_{m}=l+({\int }_{0}^{{t}_{r}}{V}_{1}{\rm{dt}}+{\int }_{{t}_{r}}^{{t}_{2}}{V}_{2}{\rm{dt}})$$

In the equation above, *S*_*m*_(m) is the stopping sight distance or safe distance headway between front vehicle i and following vehicle i+1 in the same lane, *V*_1_ (m/s) is the vehicle speed during the reaction period (m/s), *V*_2 _(m/s) expresses the vehicle’s speed when the brake pedal is pressed, ${t}_{r}$ is the reaction time, *t*_2_ (s) − *t*_*r *_(s) expresses the time required for a given effective braking distance, and *l* = 4 m is the distance between vehicles i and i + 1 at a standstill.

When considering hydroplaning, the model for the reaction process and braking process must be modified, as discussed in the following sections.

#### Modelling the reaction process

The reaction distance component of the reaction process depends on the water drag force in the horizontal direction, which is produced by the accumulation of water. Compared to the effects of this force, the effects of other forces, such as the aerodynamic drag and rolling drag, are negligible based on the low overall speed. The following equation is proposed to model the effects of the water drag force:

2 $$Fg=W{\dot{v}}_{1}={R}_{w}g$$

where *v*_1_ (m/s^2^) is the vehicle deceleration during the reaction process; *W* is the vehicle load (measured in tf); *g* = 9.8 (m/s^2^) is the acceleration due to gravity; and the water drag force *R*_*w*_ (tf) is proportional to the hydrodynamic pressure per square metre *P *(tf/m^2^) on each tire. These parameters are defined in Eqs. 3 to 5.

3 $${R}_{w}=-\,4P{S}_{V}$$

4 $$P={K}_{d}{V}^{2}$$

5 $${S}_{V}=hb$$

In the equations above, *P* (tf/m^2^) is the hydrodynamic pressure per square metre on each tire, which is proportional to the square of the vehicle speed. The hydrodynamic pressure coefficient *K*_*d*_ is equal to 0.03 (tf·s^2^/m^4^). *V* is the vehicle speed during the reaction period (m/s), *S*_*V*_ is the frontal projected area of contact between the accumulation of water and a tire (m^2^), *b* (m) is the width of a tire, and *h* is the water depth (m). Because each tire holds 25% of the total load, the result of Eq. 3 should be multiplied by four. The negative sign in Eq. 3 indicates that the water drag force opposes the vehicle speed during the reaction process. Therefore, the total deceleration can be expressed by Eq. 6.

6 $${\dot{v}}_{1}=-\,(4P{S}_{V}g)/W=-(4{K}_{d}{{\rm{V}}}^{2}{S}_{V}g)/{\rm{W}}$$

The motion and corresponding displacement with the reaction time *t*_*r*_(s) are calculated as follows:

7 $$V(t)={\int }_{0}^{{t}_{r}}\left(-4{K}_{d}{V}^{2}{S}_{V}\frac{g}{W}\right)dt=\frac{{V}_{0}}{\left(\frac{4{V}_{0}{K}_{d}{S}_{V}g}{W}\right){t}_{r}+1}$$

8 $${S}_{1}={\int }_{0}^{{t}_{r}}Vdt=\frac{W}{4{K}_{d}{S}_{V}g}\,\mathrm{ln}\left(\frac{4{V}_{0}{K}_{d}{S}_{V}g}{W}{t}_{r}+1\right),$$

where*V*_0 _(m/s) is the vehicle’s initial speed during the reaction stage. The reaction distance *S*_1_ is proportional to thereaction time *t*_*r*_. In this study, *t*_*r*_ was considered to vary with the water film depth. The reason for considering different reaction times is that in more complex scenarios, the reaction times may increase. If the water film depth increases, to ensure security, drivers require additional time to evaluate the situation and will not press the brake pedal immediately. The determination of *t*_*r*_ values will be detailed in Section “Calibration of the reaction time”.

#### Modelling the braking process

**Figure 1:**
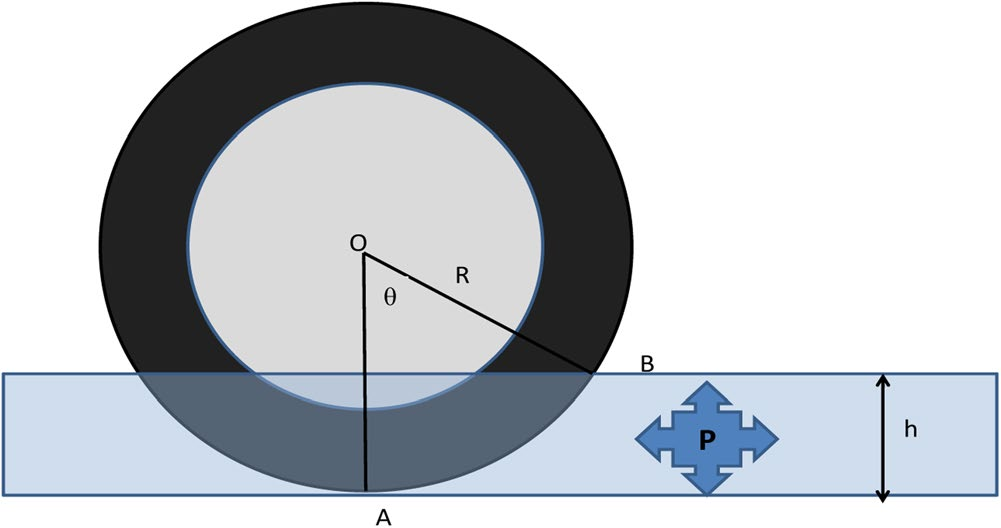
Diagram of the water pressure applied to the tire. O is the centre point of the tire. A is the intersection point between the downward vertical line from O and the ground. B is the intersection point of the tire and the surface of the water.

Braking distance estimation is a highly nonlinear problem because the tire speed profile during the braking process is a function of the braking time to be predicted. The distance component of the braking process depends on the water drag force in the horizontal direction that is produced by the accumulation of water, as well as a longitudinal friction factor (tire-to-pavement). Compared with the effects of these two forces, the effects of other factors, such as the aerodynamic drag and rolling drag, are negligible based on the low overall speed. Therefore, this study restricted the braking process to these two types of forces. The following equation is proposed to model these forces:

9 $$Fg=W{\dot{v}}_{2}={R}_{f}g+{R}_{w}g$$

where ${\dot{v}}_{2}$ (m/s^2^) is the deceleration during the braking process and *R*_*f*_  (tf) is the friction force.The components of the equation above are detailed in Eqs. 10 to 13.

10 $${R}_{f}=-\,4{f}_{0}\left(\frac{W}{4}-N\right)$$

11 $$N=P{S}_{h}$$

12 $${S}_{h}=bR\,\sin \,\theta $$

13 $$\theta ={\cos }^{-1}((R-h)/R)$$

where *f*_0_ is the tire-pavement friction coefficient under wet conditions, *N* (tf) is the lift force produced by the accumulation of water on each tire, *S*_*h *_(m^2^) is the horizontal projected area of the contact between the water surface and the tire, *R* (m) is the radius of the tire, and *θ* is the angle ∠AOB indicated in Fig. 1. In this figure, O is the centre point of the tire, A is the intersection point between the downward vertical line from O and the ground, B is the intersection point of the tire and the surface of the water, and *b* is the width of the tire rubber. The negative sign in Eq. 10 indicates that the friction force opposes the vehicle speed during the braking process.

At this point, the deceleration can be calculated using Eq. 14.

14 $${\dot{v}}_{2}=M{V}^{2}-{f}_{0}g,$$

where

15 $$M=\frac{4{K}_{d}g}{W}({f}_{0}Rb\,\sin \,\theta -{S}_{V}).$$

By using the parameter *M* Eq. 14 can be transformed into Eq. 16.

16 $$\frac{dV}{{V}^{2}-B}=Mdt,$$

Where $$B=\frac{{f}_{0}g}{M}$$.

The motion equations during the braking process differ depending on whether *M* is greater than or less than zero (Eq. 17).

17 $$\{\begin{array}{l}V=\frac{1-{e}^{D}}{1+{e}^{D}}\sqrt{B}\,(M > 0\,)\\ D=2M\sqrt{B}t+\,\mathrm{ln}|\frac{{V}_{10}-\sqrt{B}}{{V}_{10}+\sqrt{B}}|\\ V=\sqrt{-B}\,\tan \left(M\sqrt{-B}t+\arctan \frac{{V}_{10}}{\sqrt{-B}}\right)(M < 0),\end{array}$$

where *V*_10 _(m/s) is the initial speed during the braking process, which is equal to the final speed during the reaction process.

The sign of *M* is determined by the water film depth *h*, coefficient of friction *f*_0_, radius of the tire *R* (m), and other values based on Eq. 18.

$$\begin{array}{rcl}M & = & \frac{4{K}_{d}g}{W}({f}_{0}Rb\,\sin \,\theta -{S}_{V})\\  & = & \frac{4{K}_{d}gb}{W}\left({f}_{0}R\,\sin \left\{{\cos }^{-1}\left(\frac{R-h}{R}\right)\right\}-h\right)\end{array}$$

18 $${\rm{if}}\left({f}_{0}R\,\sin \left\{{\cos }^{-1}\left(\frac{R-h}{R}\right)\right\}-h\right)\ge 0,\,M\ge 0,\,{\rm{else}}\,M < 0$$

The braking time *t*_2_ is the time duration from the point at which the speed equals *V*_10_ to the point at which the speed equals zero. The corresponding equations differ depending on whether *M* is greater than or less than zero. Two boundary conditions are required to solve this equation. First, when *t*= *t*_*r*_*, V* *=* *V*_10_. Second, when *t* *= t*_2_*, V* *= 0*. Therefore, we have

19 $$\{\begin{array}{rcl}{t}_{2} & = & -\frac{1}{2M\sqrt{B}}\,\mathrm{ln}|\frac{{V}_{10}-\sqrt{B}}{{V}_{10}+\sqrt{B}}|(M > 0)\\ {t}_{2} & = & -\frac{1}{M\sqrt{-B}}\arctan \left(\frac{{V}_{10}}{\sqrt{-B}}\right)\,(M < 0)\end{array}$$

As a result, during the braking process, the displacements of *S*_2_ (m) can be expressed by Eq. 20 based on two additional boundary conditions. First, when *t* *= t*_*r*_*, S*_2_ *= 0*.Second,when *t= t*_2_*, V* *= 0*.

20 $$\{\begin{array}{rcl}{S}_{2} & = & -\frac{1}{M}\,\mathrm{ln}\left(\frac{2\times \sqrt{|\frac{{V}_{10}-\sqrt{B}}{{V}_{10}+\sqrt{B}}|}}{1+|\frac{{V}_{10}-\sqrt{B}}{{V}_{10}+\sqrt{B}}|}\right)(M > 0)\\ {S}_{2} & = & \frac{1}{M}\,\mathrm{ln}\left(\cos \left(\arctan \frac{{V}_{10}}{\sqrt{-B}}\right)\right)(M < 0)\end{array}$$

The operation of an antilock braking system and the fluid-structure interactions between a tire and water were studied^17^. The braking process of a grooved radial tire on a wet road with a 10-mm film depth was simulated at a series of discretised speeds using the commercial finite element software ABAQUS with a discretised analysis method. The automobile tire model P205/55R16 was considered for numerical simulation with an initial speed for the braking process *V*_10_ of 70 km/h, frictional coefficient (tire-ground) *f*_0_ of 0.7, and total vehicle load *W* of 1.469 tf. Table 1 lists the theoretical results from Eqs. 19 and 20 and a comparison to the braking time and distance predicted using the method proposed by the research mentioned above^17^. The agreement between the two sets of results is good. Therefore, we can use Eqs. 19 and 20 to calculate the braking distance and time under wet conditions.

**Table 1:** Comparison of estimated braking times and distances.

Item	Zang’s research	This study
Total braking time (s)	3.6	3.4
Total braking distance (m)	37.29	36.18

It lists the theoretical results from Eqs. 19 and 20 and a comparison to the braking time and distance predicted using the method proposed by the research of Zang *et al*. (2012).

**Figure 2:**
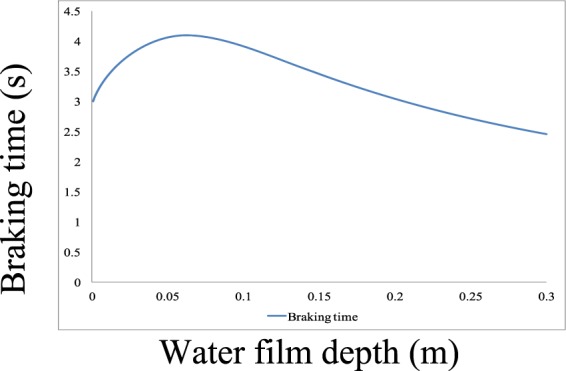
Fig. 2 shows the braking time trend according to different water film depths. When the water film depth is low, the curve shows an increasing trend. When the water film depth reaches 0.063 m, the braking time reaches its maximum value of 4.0928 s, after which it begins to decrease with increasing water film depth.

**Figure 3:**
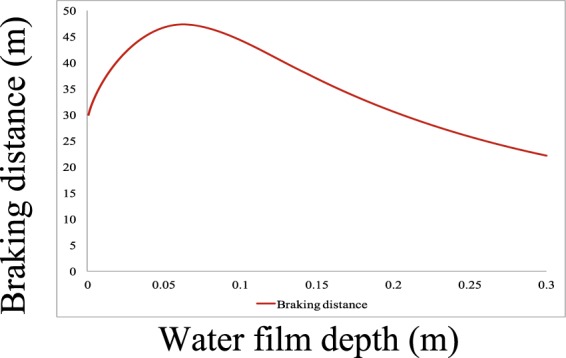
Braking distance trend according to different water film depths. When the water film depth is low, the curve shows an increasing trend. When the water film depth reaches 0.063 m, the braking distance reaches its maximum value of 47.293 m, after which it begins to decrease with increasing water film depth.

**Figure 4:**
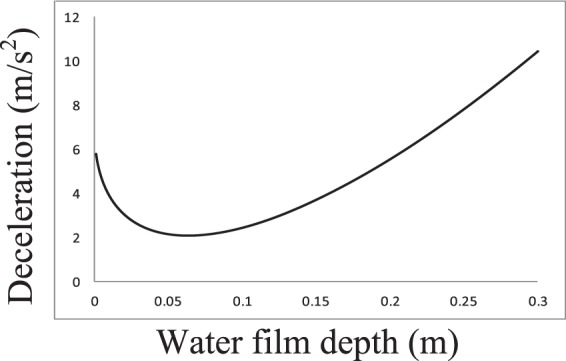
Deceleration trend according to different water film depths. When the water film depth is low, the deceleration decreases with increasing water film depth. When the water film depth reaches 0.063 m, the deceleration reaches its minimum value of 2.069 m/s^2^, after which it begins to increase with increasing water film depth.

In the real world, the braking distance and time vary with increasing water film depth. The calculation results using Eqs. 19 and 20 are presented in Figs. 2 and 3 with an initial speed *V*_10_ of 70 km/h. The deceleration results for different water film depths are presented in Fig. 4. From these figures, one can conclude that the changing tendencies of the braking time and distance are similar. When the water film depth is low, the curves show increasing trends. When the water film depth reaches 0.063 m, the braking time and distance reach their maximum values of 4.0928 s and 47.293 m, respectively, after which they began to decrease with increasing water film depth. For deceleration, when the water film depth is low, the deceleration decreases with increasing water film depth. When the water film depth reaches 0.063 m, the deceleration reaches its minimum value of 2.069 m/s^2^, after which it begins to increase with increasing water film depth. This is because when the water film depth is low, the hydrodynamic pressure *P* in the longitudinal direction plays a major role in reducing the road-tire friction coefficient, which decreases the deceleration and increases the braking time and distance. When the water film depth is high, the hydrodynamic pressure *P* in the horizontal direction plays a major role by shortening the braking process and increasing deceleration. As the water film depth approaches 0 m, the braking time approaches 3 s, and the braking distance approaches 30 m, meaning that the braking distance with a 10-mm water film depth is increased by 20% compared to that under dry conditions with an initial speed of 70 km/h, *f*_0_ = 0.7, and a tire model of P205/55R16.

### Modelling K-V curves according to different water film depths

When ignoring the differences in vehicle speeds within a traffic stream and considering the relationship between the traffic stream density and traffic spacing, the average traffic density *K *(veh/km) can be obtained based on the reciprocal of *S*_*m*_. This is a popular method in traffic engineering when deriving *K-V* relationships. The specific calculation is

21 $$K=1000/{S}_{m}$$

Because *S*_*m*_ varies with the water film depth, the *K-V* curves should also change with different water film depths.

### Case study and parameter calibration

In this section, we apply our model to a real road and attempt to construct its fundamental traffic diagram according to different water film depths.

### Data collection

The target road for this study was state road number 202.The survey was conducted on July 2009, when the city of Fukuoka, Japan was experiencing heavy rainfall. The speed limit in the target location is 40 km/h. A total of 45 min of observation was performed on site. The rainfall exceeded 100 mm/h, and the maximum depth of the water reached 20 cm during the observation period. Cameras were used to obtain vehicle and water status data simultaneously for evaluation.

### Methods for measuring the water depth and the modelling free flow speed

The method and results for measuring the free-flow speed are the same as those presented in the previous paper^16^. Sample data were collected from individual cars. First, the clock times when the front and rear of a car passed by a fixed point were measured. The free-flow speed was then obtained by dividing the car length by the difference between the two times.

**Figure 5:**
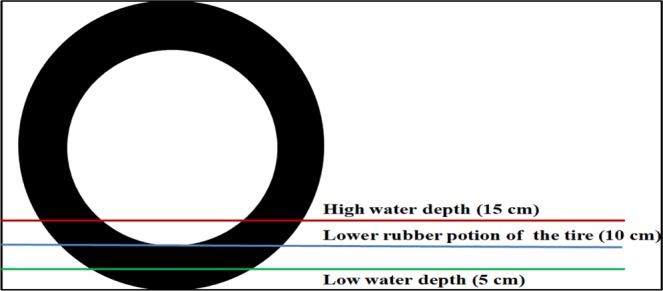
Method for evaluating the water film depth^16^. We considered 10 cm as the standard height of the lower rubber portion of a tire. If the water film depth exceeded the lower rubber portion of a tire, it was considered to be greater than 10 cm. If the depth was within the lower rubber portion of the tire, it was considered to be less than 10 cm.

For categorising observation data, it was crucial to measure the water film depth, and several water film depth (WFD) models are available. For example, a previous study^18^ proposed an analytical WFD model by simulating the dynamics of sheet flow, and this model was proven to be more accurate than the Gallaway model and PAVDRN model. The intervening factors on WFDs are ranked as rainfall intensity, pavement permeability, flow path length, flow path slope, and texture depth^18^. However, the WFD methods can predict only the water film depth, and the state of vehicle motion cannot be estimated simultaneously. Therefore, to obtain the traffic state and water film depth simultaneously, in this stage, the water film depth was obtained from video footage based on the thicknesses of the lower rubber portions of tires instead of by using any WFD method. For different types of cars, this rubber thickness varies from 9.75 cm to 12.675 cm (Table 2). Therefore, we considered 10 cm as the standard height of the lower rubber portion of a tire (blue line, Fig. 5). If the water film depth exceeded the lower rubber portion of the tire, it was considered to be greater than 10 cm. If the depth was within the lower rubber portion of the tire, it was considered to be less than 10 cm. The first situation was defined as a high water film depth. To obtain accurate measurements, we selected data with a water line located in the red line in Fig. 5 as study objects because this level represents half of the radius of a tire, making it easier to evaluate. Therefore, we considered 15 cm as the water film depth for the first condition. The second situation was defined as a low water film depth. If a water film is very thin, it cannot be identified accurately. Therefore, to improve accuracy, we selected data with a water line located at half of the height of the lower rubber portion of the tire (green line, Fig. 5) as study objects. This height is equal to 5 cm. The bounded water depth was set to 30 cm because above 30 cm, an automobile cannot operate properly. By using this method, we were able to obtain two types of depth information accurately.

**Table 2:** It shows the widths and thicknesses of different types of tires^16^. For different types of cars, this rubber thickness varies from 9.75 cm to 12.675 cm and width varies from 15.5 cm to 21.5 cm.

	Width of rubber (cm)	Thickness of rubber (cm)
Lightweight car	15.5	10.075
Small car	19.5	9.75
Medium car	19.5	12.675
Large car	21.5	11.825

**Figure 6:**
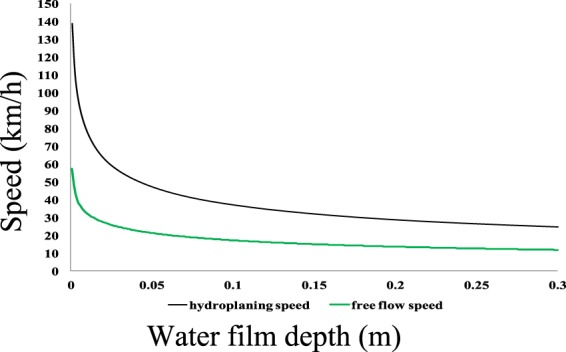
Free-flow speeds corresponding to different water depths. The trend of the hydroplaning speed *V*_*p*_ with different water film depths is plotted as a solid black curve in this figure. After adding a safety factor of 0.275, the results are presented in this figure as a solid green curve. The theoretical speed values in the 0.05-m and 0.15-m situations are 21.32 km/h and 15 km/h, respectively. These results are very consistent with the average value of the observations, with differences of −0.56% and 2.17%, respectively. We consider this speed difference to be small, meaning the model can accurately represent the free-flow speed.

From the data, the average observed free-speed values were 14.68 km/h and 21.44 km/h under high and low water film conditions, respectively. According to the type P205/55R16 tire and passenger car specifications, the total vehicle load *W* was 1.469 tf (14.4 kN).The width of a car was taken as 2 m, and the height of a car was taken as 1.5 m. The tire-road coefficient of friction under the wet condition *f*_*o*_ was set to 0.35 based on the poor driving conditions resulting from wet roads^19^. Poor conditions in this study are defined as conditions where (1) a road is covered by standing water, which results in poor surface traction, and (2) drivers are less likely to apply similar pressure to brake pedals compared to their response in dry conditions. Based on the parameters discussed above, the trend of hydroplaning speed *V*_*p*_ with different water film depths is plotted as a solid black curve in Fig. 6.The equation of calculation of *V*_*p*_ is taken from the literature^16^, and the equation is shown as Eq. 22.

22 $${V}_{p}=\sqrt{1000W({f}_{0}-\mu )/\left(\frac{1}{2g}{C}_{D}\rho A+4000{K}_{d}bh+4000{f}_{0}{K}_{d}{S}_{h}\right)}$$

where *μ* = 0.015 is the rolling resistance coefficient; *f*_0_ = 0.35 is the coefficient of friction in wet conditions; *W* is the vertical load of the car measured in tf; *C*_*D*_ = 0.4 is the aerodynamic resistance coefficient; ρ = 1.3 (kg/m^3^) is the air density; *A* is the front projected area of the car (m^2^); *g* =9.8 (m/s^2^) is the acceleration due to gravity; *K*_*d*_ = 0.03 (tf s^2^/m^4^) is the hydrodynamic pressure coefficient; *h *(m) is the water film depth; and *S*_*h *_(m^2^) is the horizontal projected area of the contact between the water surface and the tire.

The results of adding a safety factor of 0.275 are presented in Fig. 6 as a solid green curve. The theoretical speed values in the 0.05-m and 0.15-m situations are 21.32 km/h and 15 km/h, respectively. These results are very consistent with the average value of the observations, with differences of −0.56% and 2.17%, respectively. We consider this speed difference to be small, meaning the model can accurately represent the free-flow speed.

### Method for measuring the distance headway

Headway distance data were also obtained from video footage. Video extraction is a very common method for obtaining data by tracing individual vehicle movement through a specific place. In other words, each vehicle is traced in time and space as it crosses a particular place. In this study, the time at which each vehicle arrived at a fixed point was collected while processing the video footage. We could read the exact time stamps from the video footage, which guaranteed measurement precision.

The study objects were passenger cars that drove close to preceding vehicles. The samples used for measuring the distance headway were vehicles with a distance headway less than 100 m. The detailed distance headway measuring method is defined in Eqs. 23–25 and denoted in Figs. 8 and 9.

23 $${t}_{h}={T}_{h}^{i+1}-{T}_{h}^{i}$$

24 $${V}^{i+1}=\frac{{L}_{AB}^{i+1}}{{t}_{AB}^{i+1}}\times 3.6$$

25 $${S}_{m}=\frac{{V}^{i+1}}{3.6}\times {t}_{h}$$

**Figure 7:**
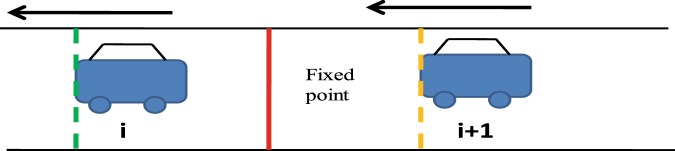
It shows the distance headway measurement method. In Eqs. 23–25, *t*_*h*_ (s) is the time headway between the front vehicle *i* and the following vehicle *i* + 1; ${T}_{h}^{i+1}$ (s) is the clock time when front vehicle *i* + *1* (position of the yellow line) passes through the red line in this figure; ${T}_{h}^{i}$ (s) is the clock time when front vehicle *i* (position of the green line) passes through the red line; *V*^*i*+1 ^(km/h) is the speed of the following vehicle; and S_m_ (m) is the distance headway between vehicles *i* and *i* + *1*.

**Figure 8:**
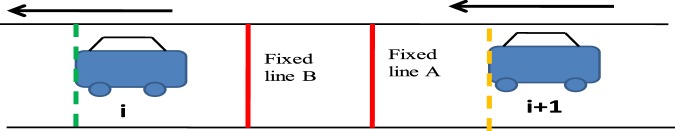
Velocity measurement method. In Eq. 23, *V*^*i*+1 ^(km/h) is the speed of the following vehicle; ${L}_{AB}^{i+1}$ (m) is the distance between the two fixed lines A and B in this figure; and ${t}_{AB}^{i+1}$ (s) is the total time required for the front of the following vehicle (yellow line) to pass through the two red lines in this figure, which is the clock time when the front of the following vehicle passes through the location of red line B minus the clock time when the front of the following vehicle passes through the location of red line A.

**Figure 9:**
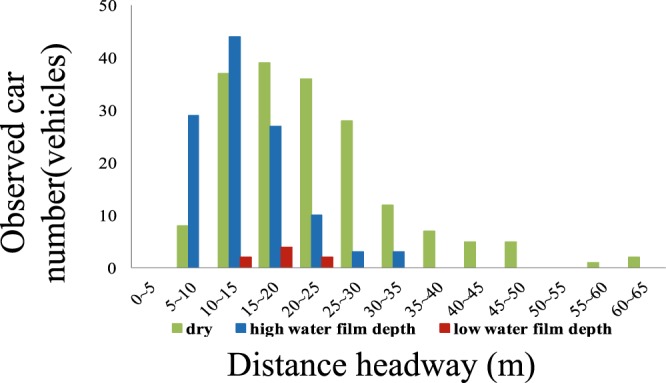
Results of the distance headway measurement. In the results for the distance headway, 116 samples were captured at a high water film depth, and 8 samples were captured at a low water film depth. To draw comparisons, we also measured values on a dry day, where 180 samples were obtained. In the high water film situation, the average distance headway was 14 m. In the low water film situation, the average value was 17 m. In the dry situation, the average value was 22.7 m.

In the equations above, ${t}_{h}$ (s) is the time headway between a front vehicle *i* and following vehicle *i* + 1; ${T}_{h}^{i+1}$ (s) is the clock time when the front of the following vehicle *i* + 1 (position of the yellow line) passes through the red line in Fig. 7; ${T}_{h}^{i}$ (s) is the clock time when the front of vehicle *i* (position of the green line) passes through the red line; ${V}^{i+1}$(km/h) is the speed of the following vehicle *i* + 1; ${L}_{AB}^{i+1}$ (m) is the distance between two fixed lines A and B (Fig. 8); $\,{t}_{AB}^{i+1}$ (s) is the total time required for the front of the following vehicle (yellow line) to pass through the two red lines in Fig. 8, which is the clock time when the front of the following vehicle passes through the location of red line B minus the clock time when the front of the following vehicle passes through the location of red line A (Fig. 8); and *S*_*m*_ (m) is the distance headway between vehicles *i* and *i* + 1.

### Results of measuring the distance headway

**Figure 10:**
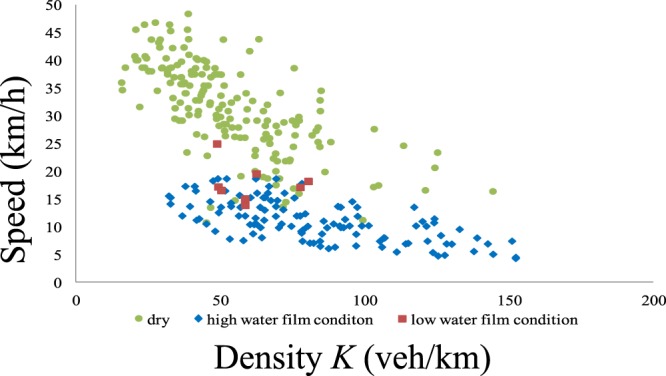
Relationship between the traffic density *K* (veh/km) and the traffic speed *V* (km/h). The relationship between the density *K* (veh/km) and the travel speed *V* (km/h) can be derived from the reciprocal of the average distance headway.

In the results for the distance headway, 116 samples were captured at a high water film depth, and 8 samples were captured at a low water film depth. To draw comparisons, we also measured values on a dry day, where 180 samples were obtained. In the high water film situation, the average distance headway was 14 m. In the low water film situation, the average value was 17 m. In the dry situation, the average value was 22.7 m. The measurement results are presented in Fig. 9. From these results, one can see that the average distance headway decreases with increasing water film depth. The relationship between the density *K* (veh/km) and the travel speed *V* (km/h) can be derived from the reciprocal of the average distance headway. This is demonstrated in Fig. 10.

### Calibration of the reaction time

Prior to calculating theoretical S_m_ values, we must determine an appropriate reaction time *t*_*r*_. This value was determined by comparing the theoretical S_m_ values calculated using Eq. 1 to the results of the distance headway under wet conditions from our observation data. From the measurement results, S_m_ with a low water film depth (h = 0.05 m) is equal to 17 m when the initial speed is 22 km/h. With a high water film depth (h = 0.15 m), S_m_ is equal to 14 m when the initial speed is 15 km/h. In our observations, the free-flow speeds were approximately 22 km/h and 15 km/h under low and high water depth conditions, respectively. Under these two conditions, the vehicles were driving at very low speeds, meaning that the differences in the distance headway were small. The reaction times were determined to be 1.6 s and 2.6 s for water film depths of 0.05 m and 0.15 m, respectively, with the parameter *l* in Eq. 1 being set to 4 m. Furthermore, we used Eq. 26 and observed distance headway of 22.7 m under dry conditions to calibrate the reaction time under dry conditions.

26 $${{\rm{S}}}_{{\rm{m}}}=\frac{{t}_{r}}{3.6}V+\frac{{V}^{2}}{2\times {3.6}^{2}\times d}+l,$$

where *d* = 6.86 m/s^2^ (*f*_0_ = 0.7) is the deceleration of a passenger car, which is equivalent to the longitudinal friction coefficient under dry conditions multiplied by the acceleration of gravity. *V* = 40 km/h is the designed speed, and *l* = 4 m is the distance between front vehicle *i* and following vehicle *i* + 1 at a standstill. By using these parameters, the reaction time *t*_*r*_ was determined to be 0.9 s when *h* = 0 m.

**Figure 11:**
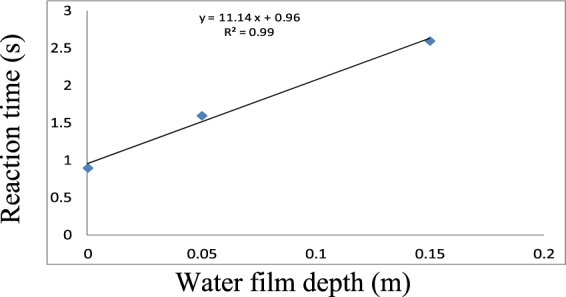
Relationship between the water film depth and the reaction time. Three reaction times were used to obtain the relationship between the water film depth and the reaction time.

The three reaction times were used to obtain the relationship between the water film depth and reaction time. The results are presented in Fig. 11.

We concluded that reaction time increases with increasing water film depth based on the linear relationship defined in Eq. 27.

27 $${t}_{r}=11.14h+0.96,$$

where *t*_*r*_ (s) is the reaction time and *h *(m) is the water film depth.

### Modelling results of the K-V and Q-V curves

**Figure 12:**
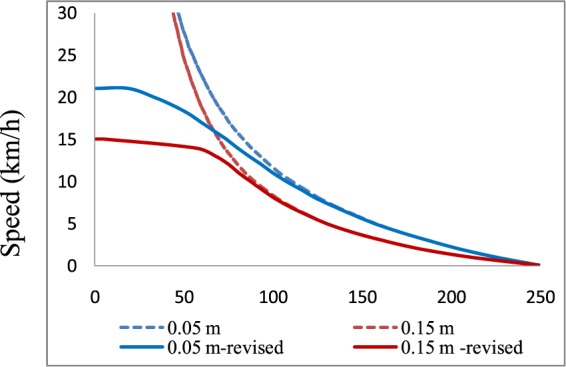
Theoretical *K-V* curves before and after adding the correction coefficient according to different water depths. The blue dotted line represents the trend before adding *β*, and the blue solid line is the trend after adding *β* for a water film depth of 0.05 m. The red dotted line is the trend before adding *β*, and the red solid line is the trend after adding *β* for a water film depth of 0.15 m.

After determining appropriate reaction times, *S*_*m*_ values could be calculated using Eq. 1. The resulting *K-V* relationship is presented in Fig. 12 (blue and red dotted lines). However, based on these results, when *S*_*m*_ is calculated using Eq. 1, regardless of the speed, if there is any hydrodynamic pressure, the car will move along the road until it stops. If friction drops to a low value, both braking problems and road slippage can occur, preventing continued vehicle operation. Another concern is that the data used for calibrating the *K-V* relationship were captured only under vehicle following conditions without considering free-flow results. Therefore, the curve shape for low vehicle densities is not consistent with practical results (when the density *K* equals zero, the speed should be equal to the free-flow speed). To overcome this issue, a correction coefficient *β* was added to the braking distance. If the friction factor *f* is smaller than a critical value, then the correction coefficient increases to infinity. Here, *β* is calculated using Eqs. 28 to 29.

28 $$\{\begin{array}{ll}\beta =\frac{\varphi {f}_{0}}{f-(1-\varphi ){f}_{0}} & (f > (1-\varphi ){f}_{0})\\ \beta =\infty  & (f\le (1-\varphi ){f}_{0})\end{array}$$

29 $$\{\begin{array}{ll}f={f}_{0}\left(\frac{\frac{W}{4}-N}{\frac{W}{4}}\right) & \,{\rm{when}}\,\left(\frac{W}{4}-N\right) > 0\\ {\rm{else}} & f=0\end{array}$$

For the equations above, *f*_0_ = 0.35 is the initial friction factor under wet conditions, and *f* is the friction factor with a water film depth *h* and velocity *V*. The equation for $\varphi$ is $\varphi$*= −A*_1_*h* + *C*, where *C* = 0.125 and *A*_1_ = 0.275 is the safety factor described in Section “Methods for measuring the water depth and the modelling free flow speed”. The calibration method for *C* is to compare the value of $1-{\rm{\varphi }}$ to the value of $\,\frac{{f}_{freeh}}{{f}_{0}}$, where *f*_*freeh*_ is the friction factor at a free-flow speed *V*_*f*_ with a water film depth *h*. In other words, the value of $1-{\rm{\varphi }}$ should be approximately equal to $\frac{{f}_{freeh}}{{f}_{0}}$. This is because if the velocity *V *at a water film depth *h* exceeds the corresponding free-flow speed *V*_*f*_, then the lift force at *V* (*N*(*V,h*)) will be greater than the lift force at *V*_*f*_ (*N*(*V*_*f*_*,h*)), leading to the friction factor *f* being less than ${f}_{freeh}=(1-\varphi ){f}_{0}$, meaning $\beta =\infty$. By using this method, we can ensure that the maximum velocity for a water film depth does not exceed the free-flow speed and that the *K-V* curves maintain a single regime.

After adding *β*, the distance during the braking process becomes

30 $${S}_{3}=\beta \,{S}_{2}.$$

Therefore,

31 $${S}_{m}=l+{S}_{1}+{S}_{3}.$$

The results after adding *β* are presented in Fig. 12. The blue dotted line in this figure represents the trend before adding *β*, and the blue solid line is the trend after adding *β* for a water film depth of 0.05 m. The red dotted line is the trend before adding *β*, and the red solid line is the trend after adding *β* for a water film depth of 0.15 m. In this figure, one can observe the following features:

1. After adding the correction parameter (solid blue and red curves), the free-flow speeds are 21 km/h and 15 km/h for water film depths of 0.05 m and 0.15 m, respectively. These values are consistent with the measured values.
2. The curve shapes at a high traffic density are nearly the same before and after adding *β* for the same water film depth, meaning that under the following car conditions, the difference in *S*_*m*_ before and after adding *β* is very small. This result proves the consistency and stability of our model.
3. After adding *β*, compared to the speed with a high water film depth, the speed with a low water film depth is greater over the full range of traffic density.

**Figure 13:**
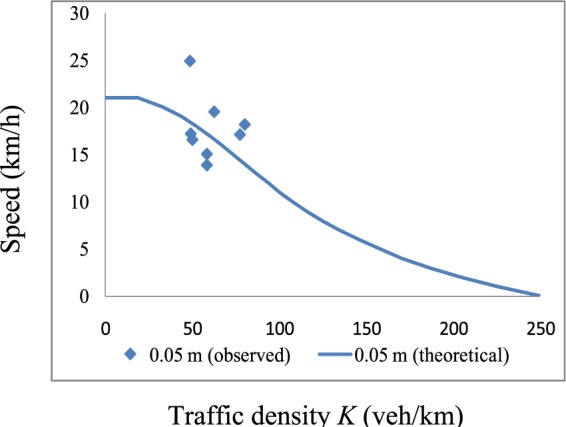
Comparison of traffic densities with a low water film depth. These results compare the theoretical *K-V* curves and measured curves for *h* = 0.05 m.

**Figure 14:**
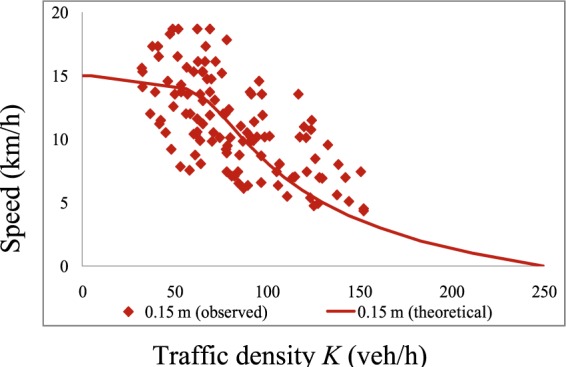
Comparison of traffic densities with a high water film depth. These results compare the theoretical *K-V* curves and measured curves for *h* = 0.15 m.

**Figure 15:**
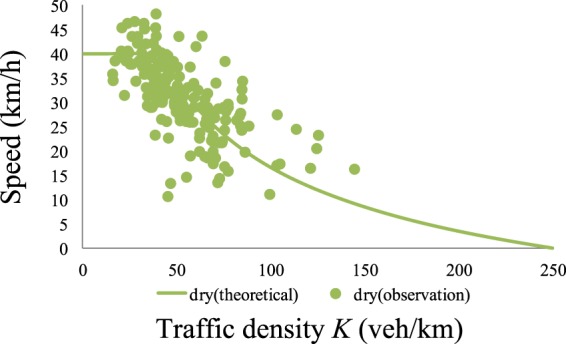
Comparison of traffic densities under dry conditions. The *K-V* curve is calculated under dry conditions using Eq. 26 with *f*_*o*_ = 0.7 and a reaction time of 0.96 s (Eq. 27). A comparison of the theoretical and measured *K-V* curves for *h* = 0 is presented.

Comparisons of the revised theoretical *K-V* curves and measured curves for *h* = 0.05 m and *h* = 0.15 m are presented in Figs. 13 and 14, respectively. From these figures, one can see that after adding *β*, the measured and theoretical curves are consistent in the low-density region. Furthermore, we obtained a *K-V* curve under dry conditions using Eq. 26 with *f*_*o*_ = 0.7 and a reaction time of 0.96 s (Eq. 27). A comparison of the theoretical and measured *K-V* curves for *h* = 0 is presented in Fig. 15. *K-S* tests were performed to determine the effectiveness of the theoretical *K-V* curves. The results are listed in Table 3.

**Table 3:** It shows the K-S test results of three conditions: low water film depth, high water film depth and dry condition. And the results proved the effective ness of the K-V curve of our model.

	D-Value	D (N, 0.2)
Low water film depth	0.0529	0.358
High water film depth	0.0573	0.0993
Dry condition	0.0076	0.07975

**Figure 16:**
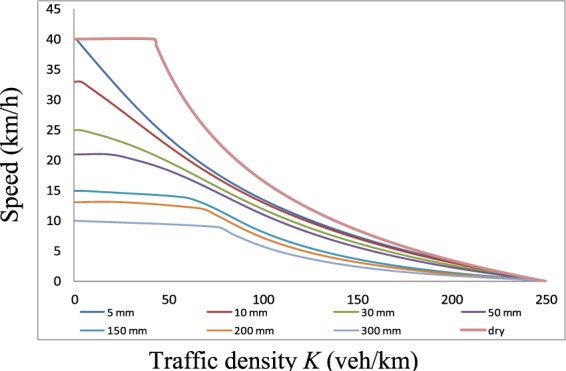
The *K-V* relationships according to different water film depths. The trends of the *K-V* curves for different water film depths are presented based on the reaction times calculated using Eq. 27.

From Table 3, the D values are all smaller than the critical values when the significance level is 0.2 in the three conditions. Therefore, we can conclude that the theoretical value is consistent with the measured value and that the *K-V* curves are suitable for reflecting practical scenarios and that the reaction time estimation is appropriate. The verification cases analysed in this section prove that accurate *K-V* curve predictions can be obtained from the proposed model for different water film depths. Additionally, the proposed analysis model can serve as a complete analytical tool for the estimation of traffic stream characteristics. The trends of the *K-V* curves for different water film depths are presented in Fig. 16 based on the reaction times calculated using Eq. 27. The results demonstrate the sensitivity of the proposed model to different water film depths. The jam density in all cases is 250 veh/km for *l* = 4 m.

**Figure 17:**
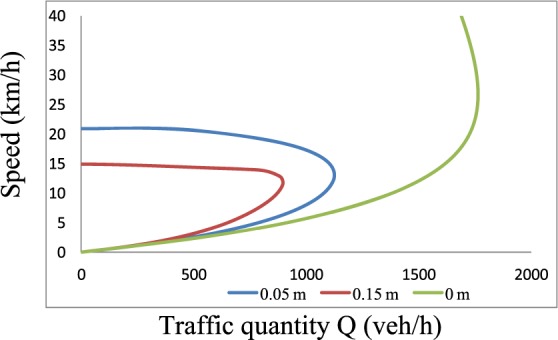
The *Q-V* curves after adding the correction coefficient. The traffic flow quantity *Q* (veh/h) can be obtained by multiplying the density *K* (veh/km) by the speed *V* (km/h). The resulting *Q-V* relationship is presented.

The traffic flow quantity *Q* (veh/h) can be obtained by multiplying the density *K* (veh/km) by the speed *V* (km/h).The resulting *Q-V* relationship is presented in Fig. 17. In this figure, the maximum traffic flows are 1125 veh/h and 899 veh/h. The corresponding critical speeds are 13 km/h and 12 km/h for water film depths of 0.05 m and 0.15 m, respectively. From the theoretical *Q-V* result under dry conditions, the capacity of the target location is 1765 veh/h, which is also shown in Fig. 17. Compared to this value, the maximum traffic flow with a high water film depth is 49% lower. The value is 36% lower with a low water film depth. This reduction in the maximum traffic flow demonstrates the road capacity losses caused by an increasing water depth.

## Evacuation simulation via an LTM considering different water film depths

**Figure 18:**
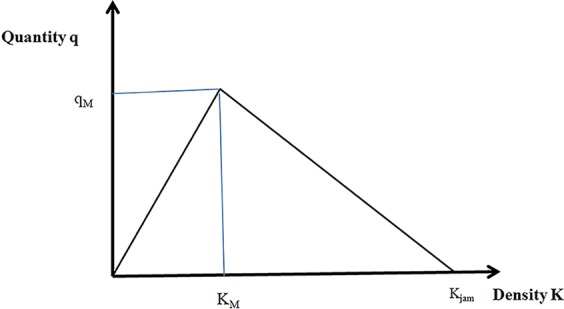
The triangular fundamental diagram. In this study, we used a traditional triangular fundamental diagram for simulation, meaning traffic states on the increasing branch of the triangular fundamental diagram (*K < k*_*M*_) hold vehicles travelling with a fixed free-flow speed *V*_*f*_. The traffic states on the decreasing branch (*K > k*_*M*_) are congested. Vehicles travel at a speed defined by $\frac{{q}_{M}}{{K}_{jam}-\frac{{q}_{M}}{{V}_{f}}}$.

In this study, traffic stream theory was simulated on a computer using an LTM^20^. The model uses macroscopic differential nonlinearity to build on Newell’s simplified theory of kinetic waves^21^. Its main components are a link model and node model that are used together to perform updates in time steps of duration *Δt*. The cumulative number of vehicles *N(x,t)* at the entrance *x*_*i*,0_ and end *x*_*i,L*_ of each link *i* is used to represent traffic. The link model is used to simulate sending and receiving flows, which indicate the number of vehicles that could potentially exit and enter the link for each time step, respectively. The node model considers the interactions of traffic at intersections to derive transition flows *G*_*ij*_(*t*). Similar to Newell’s simplified theory, the LTM uses a triangular fundamental diagram defined by three parameters: the free-flow speed (*V*_*f*_), the maximum flow (*q*_*M*_), and the jam density (*K*_*jam*_) (see Fig. 18). The LTM has been proven to provide very small numerical errors, but it supports only triangular fundamental diagrams. Some researchers have proposed methods to relax this restriction. For example, the LTM was extended to handle general concave fundamental diagrams^22^, and a concave piecewise linear fundamental diagram was used to send and receive flows to represent driving behaviour and environmental conditions accurately^23^. However, in this study, we used a traditional triangular fundamental diagram for simulation, meaning traffic states on the increasing branch of the triangular fundamental diagram (*K < k*_*M*_) held vehicles travelling with a fixed free-flow speed *V*_*f*_. The traffic states on the decreasing branch (*K > k*_*M*_) were congested. The vehicles travelled at a speed defined by $\frac{{q}_{M}}{{K}_{jam}-\frac{{q}_{M}}{{V}_{f}}}$.

### Target network and scenarios

**Figure 19:**
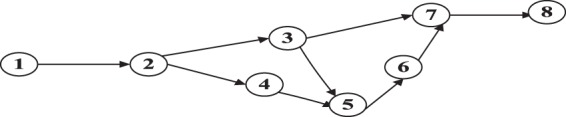
The test network of the simulation. A simple eight-node, nine-link test network was generated, which is similar to research^2^.

In this section, we present a numerical example to simulate the proposed methodology using an LTM. A simple eight-node, nine-link test network was generated, as shown Fig. 19. This network is similar to that considered by the above mentioned research^2^. The characteristics of each link are listed in Table 4. The saturation flow rate is 1765 vehicles per hour per lane, and the speed limit is 40 km/h for all links under dry conditions. Therefore, the size of the time interval Δ*t* is approximately 10 s (111 × 3.6/40 ≈ 10 s).We assume that all evacuees must evacuate the emergency area (node 1) to a safe area (node 8). The time-dependent demand characteristics are listed in Table 5. The water film depth varies with time (see Table 6). The flow characteristics, including *Q*_*max*_(*t*): the maximum number of vehicles that can flow into link *i* when the clock advances from time step *t* to *t* +1 (time interval); free-flow speed *V*_*f*_ (km/h); and *N*_*jam *_(*t*):the maximum number of vehicles that can be presented in a link at time step *t*, are listed in Table 7 (based on the results of our model). In this example, based on our model, at the jam density, we can fit 27 vehicles at 111 m. Therefore, a two-lane 111-m link can hold at most 54 vehicles.

**Table 4:** Characteristics of the example network.

Link	1–2	2–3	2–4	4–5	3–5	3–7	5–6	6–7	7–8
Length (m)	111	111	111	111	111	111	111	111	111
No. of lanes	2	1	1	1	1	1	1	1	2

The characteristics of each link are listed in Table 4, including length and number of lanes.

**Table 5:** The time-dependent demand characteristics are listed in Table 5.

Time step	1	2	3	4	……..
Demand	100	0	0	0	0

**Table 6:** Water film depth varying over time.

Time steps	Water film depth (mm)								
	Link 1–2	Link 2–3	Link 2–4	link 4–5	Link 3–5	link 3–7	Link 5–6	Link 6–7	Link 7–8
0–2	0	0	0	0	0	0	0	0	0
3–5	0	0	10	10	0	10	5	5	0
6–9	0	0	50	50	0	100	10	10	0
10–11	0	0	100	100	0	150	50	50	0
12–14	0	0	150	150	0	200	100	100	0
15–16	0	0	200	200	0	200	150	150	0
17–18	0	0	50	50	0	200	5	5	0
19–20	0	0	10	10	0	10	5	5	0
>20	0	0	0	0	0	0	0	0	0

It shows the water film depth varies with time.

**Table 7:** Flow characteristics based on the water film depth.

Water film depth(mm)	*Q*_*max*_ (veh/hour/lane)	*N*_*jam*_ (veh/km/lane)	*V*_*f*_ (km/h)
0	1765	250	40
5	1353	250	40
10	1309	250	33
50	1125	250	21
100	995	250	17
150	899	250	15
200	818	250	13

The flow characteristics, including *Q*_*max*_(*t*): the maximum number of vehicles that can flow into link *i* when the clock advances from time step *t* to *t* + 1 (time interval); free-flow speed *V*_*f*_ (km/h); and *N*_*jam*_(*t*): maximum number of vehicles that can be present in a link at a time step *t*, are listed in Table 7 (based on the results of our model).

### Analysis of results

**Figure 20:**
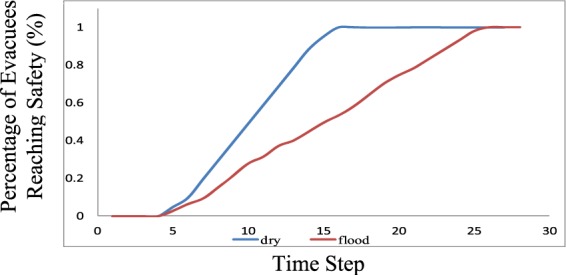
The cumulative evacuee arrival percentage. From this figure, one can see the significant impact of flow-related risks. While all evacuees arrive at the safe area under dry conditions, approximately 47% of the evacuees remain in the network under flood conditions.

The results for the test network are listed in Tables 8–11. Tables 8 and 9 list the model results under dry conditions, while Tables 10 and 11 list the results under flood conditions. When considering the water film depth risks under flood conditions, the total evacuation clearance time increases from 16 to 26 time steps. We also plotted the percentage of evacuated population versus time. In Fig. 20, one can see the significant impact of flow-related risks. While all evacuees arrive at the safe area under dry conditions, approximately 47% of the evacuees remain in the network under flood conditions. Another interesting observation is that significant evacuee rerouting occurs based on road degradation caused by water-film-depth-related risks. In the numerical example under dry conditions, the evacuees all used paths 1-2-3-7-8 and 1-2-4-5-6-7-8. Table 10 indicates that evacuees began to use link 3–5 at time step 3. This is because the water film depth at link 3–7 (belonging to route 1-2-3-7-8) increased over time, which reduced the *Q*_*max*_ and *V*_*f*_ values of the link, causing drivers to choose the route of 1-2-3-5-6-7-8.

**Table 8:** The cumulative vehicle numbers *N*(*x*_*i*,0_*, t*) at the upstream link boundary under dry conditions are listed in this table.

Time step															
Link	1	2	3	4	5	6	7	8	9	10	11	12	13	14	15
1–2	100	100	100	100	100	100	100	100	100	100	100	100	100	100	100
2–3	0	4.9	9.8	14.7	19.6	24.5	29.4	34.3	39.2	44.1	49	51	51	51	51
2–4	0	4.9	9.8	14.7	19.6	24.5	29.4	34.3	39.2	44.1	49	49	49	49	49
4–5	0	0	4.9	9.8	14.7	19.6	24.5	29.4	34.3	39.2	44.1	49	49	49	49
3–5	0	0	0	0	0	0	0	0	0	0	0	0	0	0	0
3–7	0	0	4.9	9.8	14.7	19.6	24.5	29.4	34.3	39.2	44.1	49	51	51	51
5–6	0	0	0	4.9	9.8	14.7	19.6	24.5	29.4	34.3	39.2	44.1	49	49	49
6–7	0	0	0	0	4.9	9.8	14.7	19.6	24.5	29.4	34.3	39.2	44.1	49	49
7–8	0	0	0	4.9	9.8	19.6	29.4	39.2	49	58.8	68.6	78.4	88.2	95.1	100

**Table 9:** The cumulative vehicle numbers *N*(*x*_*i,L*_*, t*) at the downstream link boundary under dry conditions are listed in this table.

Time step																
Link	1	2	3	4	5	6	7	8	9	10	11	12	13	14	15	16
1–2	0	9.8	19.6	29.4	39.2	49	58.8	68.6	78.4	88.2	98	100	100	100	100	100
2–3	0	0	4.9	9.8	14.7	19.6	24.5	29.4	34.3	39.2	44.1	49	51	51	51	51
2–4	0	0	4.9	9.8	14.7	19.6	24.5	29.4	34.3	39.2	44.1	49	49	49	49	49
4–5	0	0	0	4.9	9.8	14.7	19.6	24.5	29.4	34.3	39.2	44.1	49	49	49	49
3–5	0	0	0	0	0	0	0	0	0	0	0	0	0	0	0	0
3–7	0	0	0	4.9	9.8	14.7	19.6	24.5	29.4	34.3	39.2	44.1	49	51	51	51
5–6	0	0	0	0	4.9	9.8	14.7	19.6	24.5	29.4	34.3	39.2	44.1	49	49	49
6–7	0	0	0	0	0	4.9	9.8	14.7	19.6	24.5	29.4	34.3	39.2	44.1	49	49
7–8	0	0	0	0	4.9	9.8	19.6	29.4	39.2	49	58.8	68.6	78.4	88.2	95.1	100

**Table 10:** The cumulative vehicle numbers *N*(*x*_*i*,0_*, t*) at the upstream link boundary under flood conditions are listed in this table.

Time step																									
Link	1	2	3	4	5	6	7	8	9	10	11	12	13	14	15	16	17	18	19	20	21	22	23	24	25
**1-2**	100	100	100	100	100	100	100	100	100	100	100	100	100	100	100	100	100	100	100	100	100	100	100	100	100
**2-3**	0	4.9	9.8	14.7	19.6	24.50	29.40	34.30	39.20	44.10	49.00	53.90	58.80	61.14	61.14	61.14	61.14	61.14	61.14	61.14	61.14	61.14	61.14	61.14	61.14
**2-4**	0	4.9	8.54	12.18	15.82	18.95	22.08	25.21	28.34	31.10	33.86	36.36	38.86	38.86	38.86	38.86	38.86	38.86	38.86	38.86	38.86	38.86	38.86	38.86	38.86
**4-5**	0	0	3.64	7.28	10.92	12.49	15.62	18.75	21.88	24.08	26.84	29.15	31.65	34.15	36.15	38.42	38.86	38.86	38.86	38.86	38.86	38.86	38.86	38.86	38.86
**3-5**	0	0	1.26	2.52	3.78	5.92	8.06	10.20	12.34	14.74	17.14	19.77	22.40	25.03	25.10	25.10	25.10	25.10	25.10	25.10	25.10	25.10	25.10	25.10	25.1
**3-7**	0	0	3.64	7.28	10.92	13.68	16.44	19.20	21.96	24.46	26.96	29.23	31.50	33.77	36.04	36.04	36.04	36.04	36.04	36.04	36.04	36.04	36.04	36.04	36.04
**5-6**	0	0	0	3.76	7.52	11.16	14.80	18.44	22.08	25.21	28.34	31.10	33.86	36.62	39.12	41.62	45.38	48.51	52.15	55.79	60.69	63.96	63.96	63.96	63.96
**6-7**	0	0	0	0	3.76	6.69	10.33	13.97	17.61	18.75	21.88	24.08	26.84	29.60	31.91	34.41	38.17	41.93	45.69	49.45	54.35	59.25	63.96	63.96	63.96
**7-8**	0	0	0	2.84	6.48	9.41	15.29	21.53	27.93	31.54	37.17	40.07	44.75	49.55	53.43	58.20	64.23	70.26	74.71	78.47	83.37	88.27	93.17	98.07	100

**Table 11:** The cumulative vehicle numbers *N*(*x*_*i,L*_*, t*) at the downstream boundary under flood conditions are listed in this table.

Time step																										
Link	1	2	3	4	5	6	7	8	9	10	11	12	13	14	15	16	17	18	19	20	21	22	23	24	25	26
**1-2**	0	9.8	18.34	26.88	35.42	43.45	51.48	59.51	67.54	75.2	82.86	90.26	97.66	100	100	100	100	100	100	100	100	100	100	100	100	100
**2-3**	0	0	4.9	9.8	14.7	19.6	24.5	29.4	34.3	39.2	44.1	49	53.9	58.8	61.14	61.14	61.14	61.14	61.14	61.14	61.14	61.14	61.14	61.14	61.14	61.14
**2-4**	0	0	3.64	7.28	10.92	12.49	15.62	18.75	21.88	24.08	26.84	29.15	31.65	34.15	36.15	38.42	38.86	38.86	38.86	38.86	38.86	38.86	38.86	38.86	38.86	38.86
**4-5**	0	0	0	2.5	5	7.38	8.88	10.38	11.88	13.01	14.14	15.07	16.00	16.93	17.72	18.52	20.28	23.41	27.05	30.69	35.59	38.86	38.86	38.86	38.86	38.86
**3-5**	0	0	0	1.26	2.52	3.78	5.92	8.06	10.20	12.20	14.20	16.03	17.86	19.69	21.40	23.10	25.10	25.10	25.10	25.10	25.10	25.10	25.10	25.10	25.1	25.1
**3-7**	0	0	0	2.84	6.48	6.48	9.24	12.00	14.76	17.25	19.75	21.73	24.00	26.27	28.54	30.81	33.08	35.35	36.04	36.04	36.04	36.04	36.04	36.04	36.04	36.04
**5-6**	0	0	0	0	3.76	6.69	10.33	13.97	17.61	18.75	21.88	24.08	26.84	29.60	31.91	34.41	38.17	41.93	45.69	49.45	54.35	59.25	63.96	63.96	63.96	63.96
**6-7**	0	0	0	0	0	2.93	6.05	9.53	13.17	14.29	17.42	18.34	20.75	23.28	24.89	27.39	31.15	34.91	38.67	42.43	47.33	52.23	57.13	62.03	63.96	63.96
**7-8**	0	0	0	0	2.84	6.48	9.41	15.29	21.53	27.93	31.54	37.17	40.07	44.75	49.55	53.43	58.20	64.23	70.26	74.71	78.47	83.37	88.27	93.17	98.07	100

## Conclusions

Modelling the impact of water film depth on flooding evacuation time estimation is an important problem for public officials to avoid evacuation delays and related losses of life and property. This study aimed to identify the mechanism of water film depth that affects traffic under flood conditions. Our main contributions are summarised below.

1. We introduced a traffic characteristic model for flood evacuation planning that accounts for the water film depth, which increases the modelling accuracy.
2. We focused on the generation of reliable *K-V-h* curves according to different water film depths based on the water film mechanism of influence between tire treads and road surfaces. We constructed curves based on deceleration during the reaction and braking processes with consideration of the hydrodynamic pressure. Additionally, traffic characteristics (free-flow speed, density, and quantity) under wet conditions were quantified according to different water film depths.
3. A numerical method for estimating the total braking time and distance of vehicles driving on a wet road was developed based on a hydroplaning analysis of tires. The trends in the braking time and distance are similar. When the water film depth is low, these parameters increase. After they reach their maximum values, they begin to decrease with increasing water film depth.
4. An analytical method for estimating the reaction time was developed by comparing theoretical *S*_*m*_ values to the results of practical distance headway observations. The reaction times were determined to be 1.6 s, 2.6 s, and 0.9 s with water film depths of 0.05 m, 0.15 m, and 0 m, respectively. A linear relationship between the water film depth and the reaction time was established based on these identified points. The reaction time increases with increasing water film depth.
5. Through comparative numerical calculations, we determined that traffic capacity on a wet road is smaller than that on a dry road by 36% under low water film conditions and 49% under high water film conditions. This reduction in the maximum traffic flow indicates that the road capacity is negatively affected by floodwater.
6. Finally, the LTM was used to simulate traffic stream theory. Clearance evacuation times under both dry and flood conditions were calculated. When considering water film depth risks under flood conditions, the total evacuation clearance time increases from 16 to 26 time steps. Additionally, changes in evacuee rerouting behaviours were observed based on the link degradation caused by water-film-depth-related risks.

There are a few limitations of this study that must be addressed in the future.

1. More precise data are required to improve the accuracy of the proposed model.
2. Carefully designed experiments or investigations should be designed to derive an accurate relationship between the reaction time and water film depth.
3. The water film depth should be obtained through a more rigorous method.
